# A Sequential Optimization Sampling Method for Metamodels with Radial Basis Functions

**DOI:** 10.1155/2014/192862

**Published:** 2014-07-15

**Authors:** Guang Pan, Pengcheng Ye, Peng Wang, Zhidong Yang

**Affiliations:** School of Marine Science and Technology, Northwestern Polytechnical University, Xi'an 710072, China

## Abstract

Metamodels have been widely used in engineering design to facilitate analysis and optimization of complex systems that involve computationally expensive simulation programs. The accuracy of metamodels is strongly affected by the sampling methods. In this paper, a new sequential optimization sampling method is proposed. Based on the new sampling method, metamodels can be constructed repeatedly through the addition of sampling points, namely, extrema points of metamodels and minimum points of density function. Afterwards, the more accurate metamodels would be constructed by the procedure above. The validity and effectiveness of proposed sampling method are examined by studying typical numerical examples.

## 1. Introduction

In engineering, manufacturing companies strive to produce better and cheaper products more quickly. However, engineering systems are fairly large and complicated nowadays. In addition, design requirements are rigorous and stringent for such systems, especially multidiscipline design optimization systems such as aerospace. For example, in aircraft, the design is intrinsically a daunting optimization task often involving multiple disciplines, multiple objectives, and computation-intensive processes for product simulation. Just taking the computation challenge as an example, it is reported that it takes Ford Motor Company about 36–160 h to run one crash simulation [1], which is unacceptable in practice. Despite the fact that the capacity of computer keeps increasing, the complexity of analysis software, for example, finite element analysis (FEA) and computational fluid dynamics (CFD), seems to keep pace with computing advances [2]. To meet the challenge of increasing model complexity, design engineers are seeking new methods. As a result, metamodel which is often called surrogate model or response surface as a widely used approximation model to replace the expensive simulation is proposed and improved by researchers. In fact, in everyday life we try to save time and make predictions based on assumptions. The literature [3] describes a vivid example to strengthen understanding of metamodel. When travelling on a road we will predict the rate of turn of a bend based on the entry and surrounding landscape. Without accurately evaluating it, in our mind we are constructing metamodels using the direction of the road, its derivatives with respect to distance along the road, and local elevation information. This information is coupled with assumptions based on our experience of going round many bends in the past. Then we will calculate a suitably safe speed based on our prediction of curvature and considering a safety error. In engineering design we are also faced with different problems, but we try to do with a surrogate model essentially what we do every day in our mind: make useful predictions based on limited information and assumptions. In the past two decades, the use of metamodel [4–7] has attracted intensive attention. It is found to be a valuable tool to support a wide scope of activities in modern engineering design, especially design optimization.

Metamodeling which means the process of constructing metamodels involves two important aspects: (a) choosing a sampling method to generate sampling points and (b) choosing an approximation method to represent the data, which influence the performance of metamodels. An important research issue associated with metamodeling is how to obtain good accuracy of metamodels with reasonable sampling methods and approximation methods. Accordingly, sampling methods and approximation methods are intensively studied in recent years. As one of the most effective approximation methods, radial basis functions (RBF) [8–10] interpolation has been gained popularity for model approximation because of their simplicity and accurate results for interpolation problems. RBF is becoming a better choice for constructing metamodels or finding the global optima of computationally expensive functions by using a limited number of sampling points. Other types of approximation methods including Kriging [11], multivariate adaptive regression splines (MARS) [12], response surface methodology (RSM) [13], and support vector machines (SVM) [14], and so forth, are discussed broadly as well. Mullur et al. proposed an improved form of the typical RBF approach, that is, extended radial basis functions (E-RBF) which offers more flexibility in the metamodel construction process and provides better smoothing properties. In general, it is expected to yield more accurate metamodels than the typical RBF. So this paper uses E-RBF to construct the metamodels.

The sampling method is another important factor of affecting accuracy for a given metamodel. The sampling method can be divided into the classical sampling method, “space-filling” sampling method, and sequential sampling method according to the type. Classic sampling methods are developed from design of experiments (DOE). These methods focus on planning experiments and tend to spread the sample points around boundaries of the design space for eliminating random error. The classical experiment designs contain alphabetical optimal design [15], factorial or fractional factorial design [16], Box-Behnken [17], central composite design (CCD) [18], and so forth. However, Sacks et al. [19] stated that classic experiment designs can be inefficient or even inappropriate for deterministic optimal problems. Jin et al. [20] confirmed that experiment designs for deterministic computer analyses should be spaced filling. The space-filling sampling methods which are correspondingly more often used in the literature are Latin hypercube design (LHD) [21], orthogonal arrays design [22], Hammersley sequences (HS) [23], and uniform designs [24]. The sampling methods above are generated all at once, or, in other words, at one stage. It is difficult for the one-stage sampling methods to forecast the number of sampling points. On the contrary, the sequential sampling approach generates sampling points one after another according to the particular criteria instead of generating all points at once. In sequential sampling technique, the new sampling points are added to the sample by taking the advantage of information gathered from the existing (earlier created) metamodel and then corresponding response surface is updated. Therefore, the sequential sampling recently has gained popularity for its advantages of flexibility and adaptability over other methods. Jin et al. [25] stated that sequential sampling allows engineers to control the sampling process and it is generally more efficient than one-stage sampling. Deng et al. [26] proposed a sequential sampling design method based on Kriging. Wei et al. [27] proposed a sequential sampling method adopting a criterion to determine the optimal sampling points, which maximized the value of the product of curvature and square of minimum distance to other design sites. Kitayama et al. [28] presented a sequential approximate optimization (SAO) algorithm using the RBF network with sequential sampling methods. A novel sequential sampling method based on the maximin distance criterion was proposed by Zhu et al. [29].

In this paper, a new sequential optimization sampling method with extended radial basis functions is proposed. In order to utilize the geometrical feature of metamodels, extrema points of the response surface, as new optimization sampling points, are added to the sample. Through using the metamodeling functions constructed by extended radial basis functions, the extrema points of metamodels can be achieved expediently. Moreover, an effective function [28, 30] called the density function for determining the sparse region in the design variable space is considered. The density function constructed by using the RBF is to discover a sparse region in the design space. It is expected that the addition of sampling points in the sparse region will improve the accuracy of approximation model. Thus, a new metamodeling algorithm integrating a sequential optimization sampling method is presented. To illustrate the accuracy and efficiency of the proposed algorithm, the measure performance and several numerical examples will be tested.

The remainder of this paper is organized as follows. In next section, the RBF and E-RBF are described briefly. In Section 3, a new sequential optimization sampling method is proposed. In addition, the density function [28, 30] is introduced. In Section 4, the numerical examples, assessment measures, test results and discussions, and so forth will be provided. The last section is the closure of the paper where we summarize the important observations made from our study.

## 2. Radial Basis Functions

### 2.1. Learning of Classical Radial Basis Functions

The RBF metamodel was originally developed by Hardy [31] in 1971 to fit irregular topographic contours of geographical data. It has been known tested and verified for several decades and many positive properties have been identified. Mullur and Messac [32] made radial basis functions more flexible and effective by adding so-called nonradial basis functions. Krishnamurthy [33] added a polynomial to the definition of RBF for improving the performance. Wu [34] provided criteria for positive definiteness of radial functions with compact support which produced series of positive definite radial functions.

**Figure 1:**
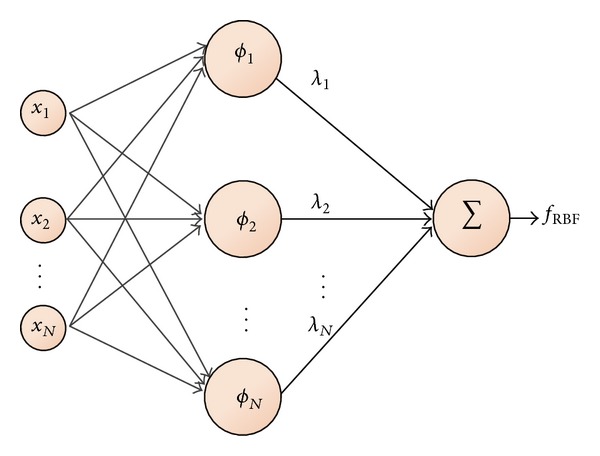
Three-layer feed-forward RBF network.

An RBF network is a three-layer feed-forward network shown in Figure 1. The output of the network $\hat{f}\left(x\right)$, which corresponds to the response surface, is typically given by

(1) $$\begin{array}{c} \hat{y}=\hat{f}\left(x\right)=\overset{N}{\underset{i=1}{\sum }}\lambda_{i}\phi \left(||x-x_{i}||\right)=\Phi \cdot \lambda , \end{array}$$

where *N* is the number of sampling points, **x** is a vector of design variables, **x**_*i*_ is a vector values of design variables at the *i*th sampling point, Φ = [*ϕ*_1_, *ϕ*_2_,…, *ϕ*_*N*_]  (*ϕ*_*i*_ = *ϕ*(||**x** − **x**_*i*_||)),  ***λ*** = [*λ*_1_,*λ*_2_,…,*λ*_*N*_]^*T*^,  ||**x** − **x**_*i*_|| is the Euclidean norm, *ϕ* is a basis function, and *λ*_*i*_ is the coefficient for the *i*th basis function. The approximation function $\hat{y}$ is actually a linear combination of some RBF with weight coefficients *λ*_*i*_. The most commonly used radial classical radial functions are listed in Table 1. The radial basis functions multiquadric and Gaussian are the best known and most often applied. The multiquadric is nonsingular and simple to use [35]. Hence, radial basis function multiquadric is used in this paper.

### 2.2. Learning of Extended Radial Basis Functions

**Figure 2:**
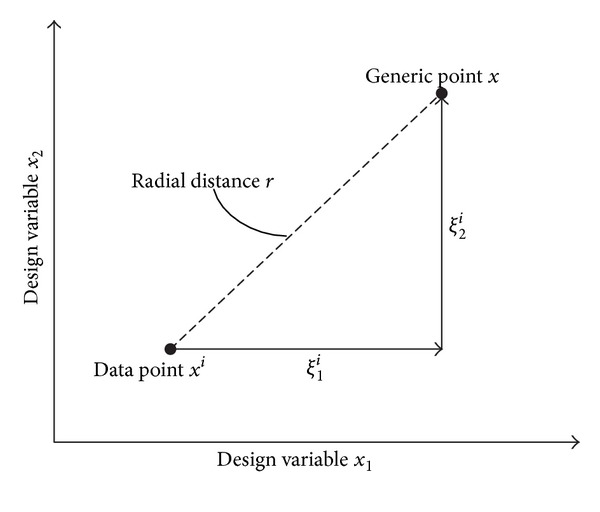
Definition of coordinate *ξ*.

**Figure 3:**
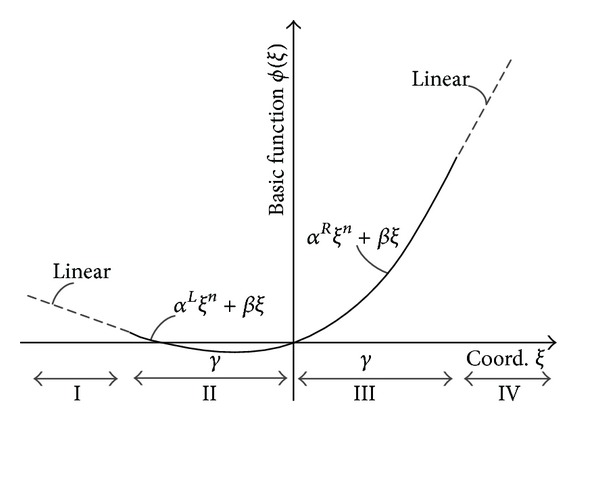
Nonradial basis functions.

The extended radial basis functions approach is a combination of radial and nonradial basis functions which incorporate more flexibility in the metamodels by introducing additional degrees of freedom in the metamodels definition. It provides better smoothing properties, and, in general, it is expected to yield more accurate metamodels than the typical RBF. Mullur and Messac [32, 36] found that the E-RBF approach results in highly accurate metamodels compared to the classical RBF and Kriging. Under the E-RBF approach, the approximation function takes the form:

(2) f^(x)=∑i=1Nλiϕ(||x−xi||)+∑i=1N∑j=1n{αijLψL(ξij)+αijRψR(ξij)+βijψβ(ξij)},

where *n* is the number of design variables; *α*_*ij*_^*L*^, *α*_*ij*_^*R*^, *β*_*ij*_ are coefficients to be determined for given problems; *ψ* ^*L*^, *ψ* ^*R*^, *ψ* ^*β*^ are components of the so-called nonradial basis functions defined in Table 2. Nonradial basis functions are functions of *ξ*_*i*_^*j*^, which is the coordinate vector of a generic point *x* in the design space relative to a data point *x*_*i*_, defined as *ξ*_*i*_ = *x* − *x*_*i*_. Thus, *ξ*_*i*_^*j*^ is the coordinate of any point *x* relative to the data point *x*_*i*_ along the *j*th dimension. The difference between the Euclidean distance *r* used in RBF and the relative coordinates *ξ* used for N-RBF for a two-dimensional case is depicted in Figure 2. Four distinct regions (I–IV) are depicted in Figure 3, each corresponding to a row in Table 2.

In matrix notation, the metamodel defined in (2) can be written as

(3) $$\begin{array}{c} \left[A\right]\left\{\lambda \right\}+\left[B\right]{\left\{{\left(\alpha^{L}\right)}^{T}{\left(\alpha^{R}\right)}^{T}\beta^{T}\right\}}^{T}=\left\{f\right\}. \end{array}$$

Equation (3) can be compactly written in matrix form as

(4) $$\begin{array}{c} \left[\overset{-}{A}\right]\left\{\overset{-}{\alpha }\right\}=\left\{f\right\}, \end{array}$$

where $\left[\overset{-}{A}\right]=\left[AB\right]$, $\left\{\overset{-}{\alpha }\right\}={\left\{\lambda^{T}{\left(\alpha^{L}\right)}^{T}{\left(\alpha^{R}\right)}^{T}\beta^{T}\right\}}^{T}$, and *f* = *f*(*x*_*i*_).

The coefficients $\overset{-}{\alpha }$ can be evaluated by using the pseudoinverse approach to solve the underdetermined system of (4) as follows:

(5) $$\begin{array}{c} \left\{\overset{-}{\alpha }\right\}={\left[\overset{-}{A}\right]}^{+}\left\{f\right\}, \end{array}$$

where ${\left[\overset{-}{A}\right]}^{+}$ denotes the pseudoinverse of $\left[\overset{-}{A}\right]$.

After obtaining the coefficients $\overset{-}{\alpha }$ using E-RBF, one can evaluate the metamodels to construct response surface using (2). The resulting metamodel is one that is guaranteed to be convex and highly accurate [32, 36]. In the following section, a series of mathematical examples are approximated by E-RBF based on the sampling method proposed in next section.

In this section, we introduce the metamodel approach E-RBF briefly due to space limitations. A more complete description and discussion are presented in articles [32, 36].

## 3. The Sequential Optimization Sampling Method

As it is known, the metamodels are approximation models of real models which are commonly complex and unknown. The accuracy of metamodels owes to the approximation methods and sampling methods primarily [37]. The sampling method proposed in this paper includes two parts. The first part is the procedure of adding optimization sampling points and the second part is the procedure of adding points of sparse regions [28, 30].

### 3.1. The Optimization Sampling Points

The optimization sampling points should have two important properties as follows: adaptive and sensitive. The focus on sampling should shift to how to generate a reasonable number of sampling points intelligently so that the metamodel can reflect the real “black-box functions” in areas of interest. General knowledge tells us that sampling points at the site of valley and peak of the response surface would improve the accuracy at the greatest extent. The valley and peak are extrema points of functions.

In mathematics, the points which are the largest or smallest within a given neighborhood are defined extrema points. McDonald et al. [38] found the radial basis functions models created with (1) are twice continuously differentiable when employing multiquadric function as basis function for all *c* ≠ 0. The first approximation model is constructed with initial sampling points through using radial basis functions. Thus the function evaluations, analytic gradients, and the Hessian matrix of second partial derivatives can be obtained from the initial functions.

Considering the RBF model with *n* dimensions in (1), the gradients of the equation are

(6) $$\begin{array}{c} \frac{\partial \hat{f}}{\partial x}=\overset{N}{\underset{i=1}{\sum }}\frac{\lambda_{i}\phi^{\prime }\left(r_{i}\right)}{r_{i}\left(x\right)}{\left(x-x_{i}\right)}^{T}, \end{array}$$

where *ϕ*′(*r*_*i*_) = ∂*ϕ*/∂*r*_*i*_, *r*_*i*_(**x**) = ||**x** − **x**_*i*_||. For multiquadric RBF model, *ϕ*(*r*_*i*_) = (*r*_*i*_^2^+*c* ^2^)^1/2^,  *ϕ*′(*r*_*i*_) = *r*_*i*_(*r*_*i*_^2^+*c* ^2^)^−1/2^.

The Hessian matrix can be calculated from (6) as

(7) H(x)=∂2f^∂x2=∑i=1Nλiri(x)[ϕ′(ri)·I+(ϕ′′(ri)−ϕ′(ri)ri(x))](x−xi)T.

Similarly, *ϕ*′′(*r*_*i*_) = ∂^2^ *ϕ*/∂*r*_*i*_^2^. For multiquadric RBF model, *ϕ*′′(*r*_*i*_) = *c* ^2^(*r*_*i*_^2^+*c* ^2^)^−3/2^. *I* is a unit vector.

Let (6) be equal to zero and solve

(8) $$\begin{array}{c} \frac{\partial \hat{f}}{\partial x}=0,\;x=\left(x_{1},x_{2},\ldots ,x_{N}\right). \end{array}$$

**Figure 4:**
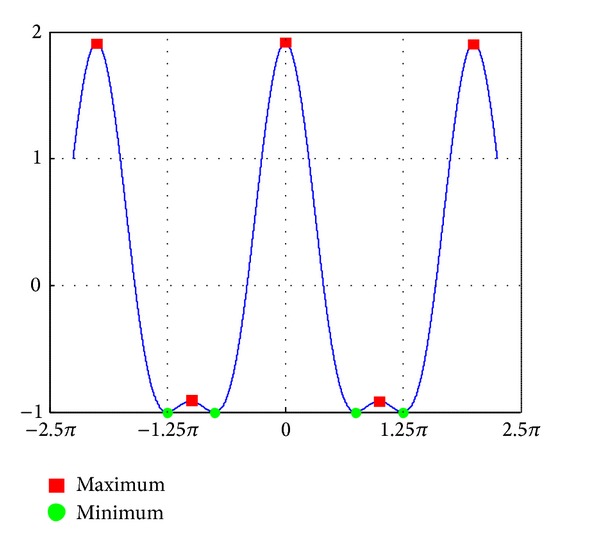
Extrema points in 2D.

**Figure 5:**
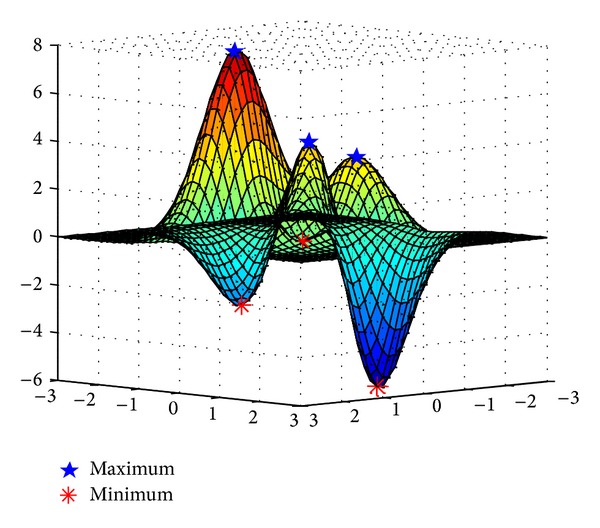
Extrema points in 3D.

Then, the critical point **x**_*e*_ can be obtained. Upon substitution of the critical point **x**_*e*_ into Hessian matrix, we can judge the definition of the matrix *H*(**x**_*e*_). The critical point is maximum or minimum when the matrix is definite positive or definite negative. Figures 4 and 5 separately show the extrema points in the case of 2D, 3D. The red square and blue pentagon indicate maxima points; meanwhile, the green dot and red asterisk indicate minima points. Once an approximation model has been created, we can obtain coordinates of extrema points.

### 3.2. Density Function with the RBF

It is necessary to add new sampling points in the sparse region for global approximation. To achieve this, a new function called the density function which is proposed by Kitayama et al. [28, 30] is constructed using the RBF network in this paper. The aim of the density function is to discover a sparse region in the design space. This density function generates local minima in the sparse region, so that the minimum of this function can be taken as a new sampling point. The addition of new sampling points in the sparse region will improve the accuracy of approximation model and help to find the global minimum of metamodels.

To explore the sparse region, every output *f*of the RBF network is replaced with +1. Let *N* be the number of sampling points. The procedure for constructing the density function is summarized as follows.

(1) The output vector *f* ^*D*^ is prepared at the sampling points:
  (9) $$\begin{array}{c} f^{D}={\left(1,1,\ldots ,1\right)}_{N*1}^{T}. \end{array}$$
(2) The weight vector *λ*_*D*_ of the density function *D*(*x*) is calculated as follows:
  (10) $$\begin{array}{c} \lambda^{D}={\left(\Phi^{T}\Phi +\Delta \right)}^{-1}\Phi^{T}f^{D}, \end{array}$$
 where
  (11) Φ=[ϕ1(x1)ϕ2(x1)⋯ϕN(x1)ϕ1(x2)ϕ2(x2)⋯ϕN(x2)⋮⋮⋱⋮ϕ1(xN)ϕ2(xN)⋯ϕN(xN)],Δ=10−3×[10⋯001⋯0⋮⋮⋱⋮00⋯1]N∗N.
(3) The addition of sampling point *x* ^*D*^ in the sparse region is explored as the global minimum of density function with the RBF:
  (12) $$\begin{array}{c} D\left(x^{D}\right)=\overset{N}{\underset{j=1}{\sum }}\lambda_{j}^{D}\phi_{j}\left(x^{D}\right)⟶\min. \end{array}$$

### 3.3. Summary of the Proposed Algorithm

**Figure 6:**
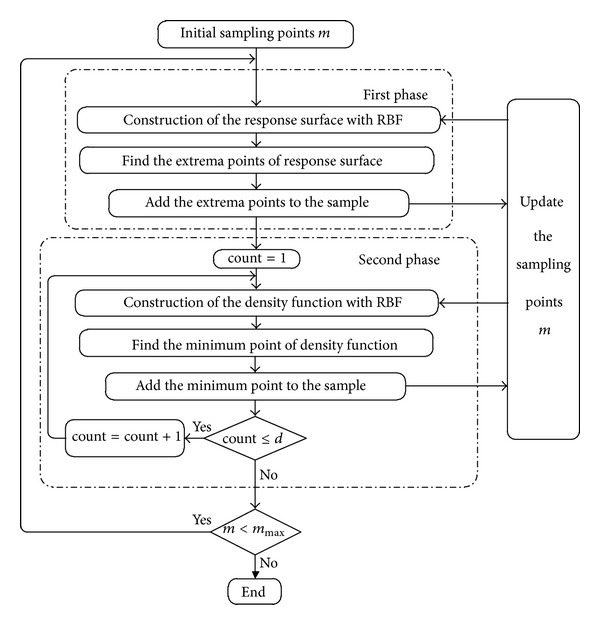
Proposed algorithm.

The detailed algorithm for sequential optimization sampling method with RBF network is described below.  Figure 6 shows the proposed algorithm. The proposed algorithm is roughly divided into two phases. The first phase is used to construct the response surface and add the extrema points of response surface as new sampling points. The second phase is used to construct the density function and add the minimum of the density function as a new sampling point. These two phases are described particularly as follows.

First phase: *m* initial sampling points are generated using the Latin hypercube sampling design. All functions are evaluated at the sampling points, and the response surface $\hat{f}\left(x\right)$ is constructed. The extrema points of response surface can then be found and directly taken as the new sampling points. The more accurate metamodel would be constructed by repeating the procedure of adding extrema points to the sample adaptively and sequentially.

Second phase: the density function is constructed to find the sparse region. The minimum point of the density function is taken as a new sampling point. This step is repeated while a terminal criterion (count ≤ *d*) is satisfied. Parameter *d* controls the number of sampling points obtained by the density function. Kitayama et al. [28] advised *d* = int⁡(*n*/2). In this paper, parameter *d* = int⁡(*n*/2) + 1, where int⁡( ) represents the rounding-off. The new sampling point would be gained if the parameter count is less than *d*, and it is increased as count = count + 1.

The terminal criterion of the integrated algorithm is determined by the maximum number of sampling points *m*_max⁡_. If the number of sampling points is less than *m*_max⁡_, the algorithm proceeds. Otherwise, the algorithm is terminated. In the algorithm, the response surface is constructed repeatedly through the addition of the new sampling points, namely, extrema points and minimum point of density function. Afterwards, the more accurate metamodel would be constructed by repeating the procedure of adding points to the sample adaptively and sequentially.

## 4. Numerical Examples

In this section, we would test the validity of the proposed sampling method through some well-known numerical examples and one engineering optimization problem. All these functions are approximated with the E-RBF model. The response surfaces are constructed through one-stage sampling methods (i.e., LHD and RND) and sequential sampling methods (i.e., CV [25], Kitayama et al. [28], SLE [29], and SOSM proposed in this paper) with the same sampling size. In order to visualize the comparison between approximated model and actual model, two design variables of numerical examples listed in Table 3 are tested.

### 4.1. Sampling Strategies

Two types of sampling methods used in this paper separately are one-stage sampling methods and sequential sampling methods. One-stage sampling methods include Latin hypercube sampling design (LHD) and Random sampling design (RND). Sequential sampling methods include cross-validation sampling method (CV) [25], sequential sampling method proposed by Kitayama et al. [28] termed KSSM in this paper, successive local enumeration sampling method (SLE) [29], and sequential optimization sampling method (SOSM) proposed in this paper. Every metamodel is constructed for 20 times with the same sampling method in this paper. For functions 1 and 2, we set *m*_max⁡_ = 25. For functions 4 and 5, we set *m*_max⁡_ = 36. For functions 3 and 6, we generate 28 and 40 sampling points separately.

### 4.2. Selection of Parameters

For the E-RBF approach, we set *c* = 1, which is a prescribed parameter for the multiquadric basis functions, for all of the examples. The parameter *γ* is set equal to approximately 1/3 of the design domain size. Mullur and Messac [32] investigated that the results were not unduly sensitive to *γ*. Another parameter *η* is set equal to 2 for all numerical examples.

### 4.3. Metamodel Accuracy Measures

Generally speaking, an E-RBF response surface passes through all the sampling points exactly. Therefore it is impossible to estimate the accuracy of an E-RBF model with sampling points. To measure the accuracy of the resulting metamodels, we can use additional testing points to evaluate the accuracy of the model via standard error measure: root-mean-squared error (RMSE). The smaller the value of RMSE is, the more accurate the response surface will be. The error measure is defined as

(13) $$\begin{array}{c} \text{RMSE}=\sqrt{\frac{\sum_{k=1}^{K}{\left[f\left(x_{k}\right)-\hat{f}\left(x_{k}\right)\right]}^{2}}{K}}, \end{array}$$

where *K* is the number of additional testing points generated by grid sampling method (32∗32 for all the examples). *f*(**x**_*k*_) and $\hat{f}\left(x_{k}\right)$ are the true function value and predicted metamodel value at the *k*th testing point **x**_*k*_, respectively.

In addition to the preceding RMSE, we also calculate the normalized root-mean-squared error (NRMSE) as follows:

(14) $$\begin{array}{c} \text{NRMSE}=\sqrt{\frac{\sum_{k=1}^{K}{\left[f\left(x_{k}\right)-\hat{f}\left(x_{k}\right)\right]}^{2}}{\sum_{k=1}^{K}{\left[f\left(x_{k}\right)\right]}^{2}}}*100\%. \end{array}$$

RMSE only calculates the error of functions themselves. However, NRMSE allows comparison of the metamodel error values with regard to different functions.

In engineering problems, global minimum is required generally. So we employ the simulated annealing (SA) [39] to calculate the global minimum ${\hat{f}}_{\min}$ based on the ultimate metamodel. The actual global minimum *f*_min⁡_ are listed in Table 3.

### 4.4. Results and Discussions

In this section, we discuss the results obtained after constructing metamodels through six various sampling methods using the assessment measure described above. As mentioned in Section 4.1, 20 procedures are conducted for each sampling method and therefore there are twenty sets of accuracy results for each sampling method. The advantages and validity of sequential optimization sampling method (SOSM) are tested in comparison with one-stage sampling methods and sequential sampling methods separately below. In addition, SOSM is used to solve a typical mechanical design optimization problem.

#### 4.4.1. Comparison of the Performance between SOSM and One-Stage Sampling Methods

In this part, two classical one-stage sampling methods LHD, RND and SOSM are used to construct metamodels. The accuracy of metamodels and global minimum of functions are obtained and managed. The error measures RMSE and NRMSE and global minimum summarized in Table 4 are average values.

From the Table 4, the RMSE and NRMSE of metamodels using sampling method SOSM are smaller than the other two one-stage sampling methods for functions 1–5. For function 6, the values are close. The RND, as expected, performs poorly. That is, metamodels based on sampling method SOSM may provide a better fit to actual functions. As seen in Table 4, on exploring global minimum ${\hat{f}}_{\min}$ of metamodels, SOSM is superior to LHD and RDN through all numerical examples compared to the actual global minimum *f*_min⁡_. In particular, the SOSM can almost find the global minimum of all numerical examples at every turn. However, LHD and RND perform fairly poorly, particularly in the success rate which will be depicted in Figure 9.

**Figure 7:**
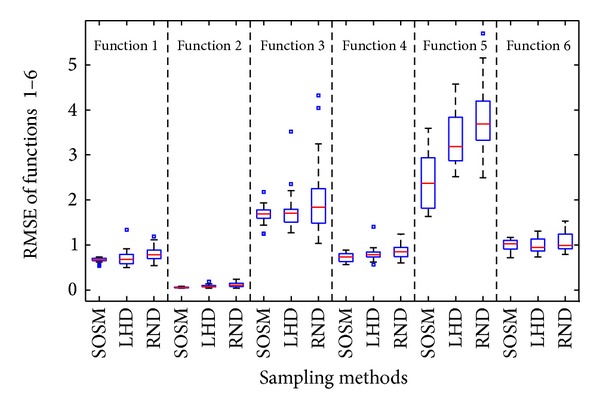
Assessment of metamodels: RMSE between SOSM and one-stage sampling methods.

**Figure 8:**
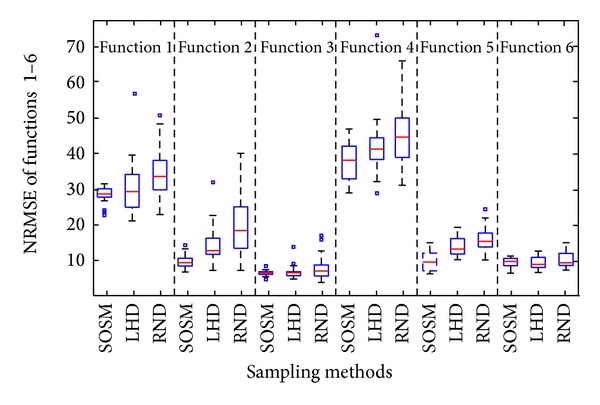
Assessment of metamodels: NRMSE between SOSM and one-stage sampling methods.

**Figure 9:**
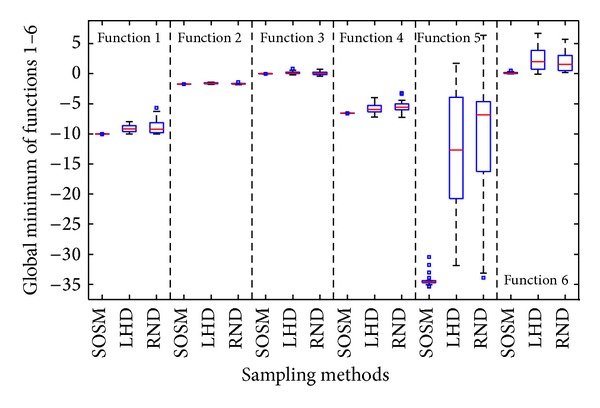
Assessment of metamodels: Global minimum between SOSM and one-stage sampling methods.

The mean results from Table 4 cannot represent the advantages and disadvantages of different sampling methods adequately. Thus, statistical graphics, that is, boxplots, are used to show the deviations of the accuracy and global minimum of each metamodel. In descriptive statistics, boxplot is a convenient way of graphically depicting groups of numerical data through their quartiles. The center line of each boxplot shows the 50th percentile (median) value and the box encompasses the 25th and 75th percentile of the data. The leader lines (horizontal lines) are plotted at a distance of 1.5 times the interquartile range in each direction or the limit of the data (if the limit of the data falls within 1.5 times the interquartile range). The data points outside the horizontal lines are shown by placing a sign (“□”) for each point. The twenty times of metamodels accuracy results (RMSE and NRMSE) and global minimum *f*_min⁡_ with sampling methods, that is, SOSM, LHD, and RND, are illustrated in Figures 7–9 with the help of boxplots.

From the results shown in Figures 7 and 8, it is found that the median values of RMSE and NRMSE are smaller compared to LHD and RND for functions 1–5. For function 6, the median values of RMSE and NRMSE are a little larger, but very close. In addition, the box size of RMSE and NRMSE based on SOSM is the shortest for all functions except for functions 4 and 5. The difference of sizes is small. It is clear that a small size of the box suggests small standard deviation of results. High standard deviation of results suggests large uncertainties in the predictions and low standard suggests low uncertainty on the contrary. The points outside the horizontal lines indicate that the results of experiments are terrible. Above all, either mean values or median values and box sizes of results are taken into account, the accuracy of metamodels with sampling method SOSM is better.

Figure 9 depicts the boxplot of global minimum ${\hat{f}}_{\min}$ obtained for twenty numerical experiments. It is obvious from Figure 9 that the median value of SOSM is probably equal to the actual global minimum *f*_min⁡_. Meanwhile, the small sizes of boxes imply small standard deviation, which is also reflected by small differences between the mean and median values. The standard deviation of global minimum is one of the important factors for evaluating the robustness of algorithm. Therefore, smaller standard deviation of results implies the robustness of the algorithm. It is clear from Figure 9 that the SOSM is a more robust sampling method than the other two sampling techniques under the parameter settings employed in this paper. The success rate is bad comparing the actual global minimum from Table 4 with the distribution of global minimum of metamodels based on sampling methods LHD and RND from Figure 9. In other words, SOSM plays a perfect role in finding the global minimum of metamodels.

**Figure 10:**
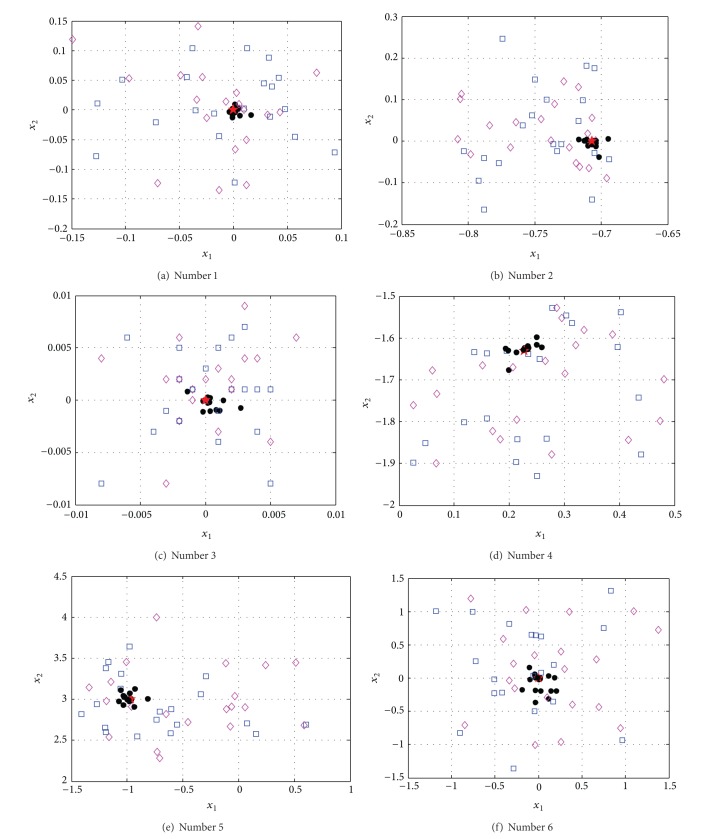
Global minima points of functions 1–6. (a)–(f) show the global minima points for functions 1–6 separately. The red pentagon indicates the actual global minimum point. The black dots indicate the global minima points of metamodels based on sampling method SOSM. The blue square indicates the global minima points of metamodels based on sampling method LHD. The magenta diamond indicates the actual global minima points of metamodels based on sampling method RND.

**Figure 11:**
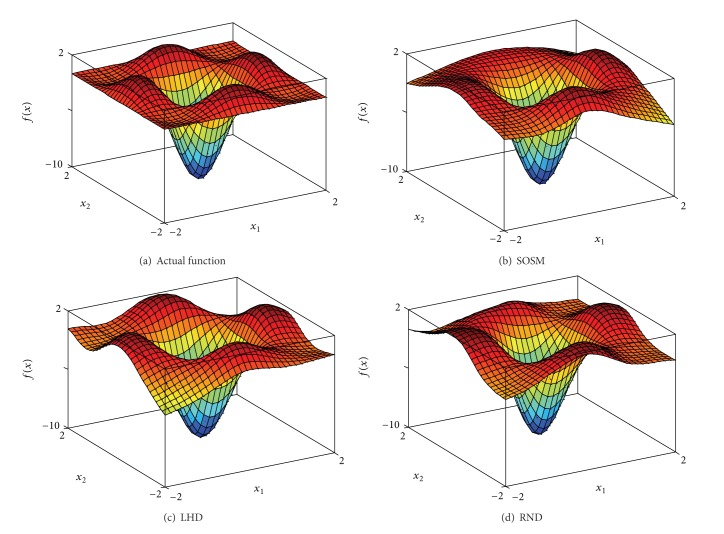
Function 1: actual and metamodel surface.

**Figure 12:**
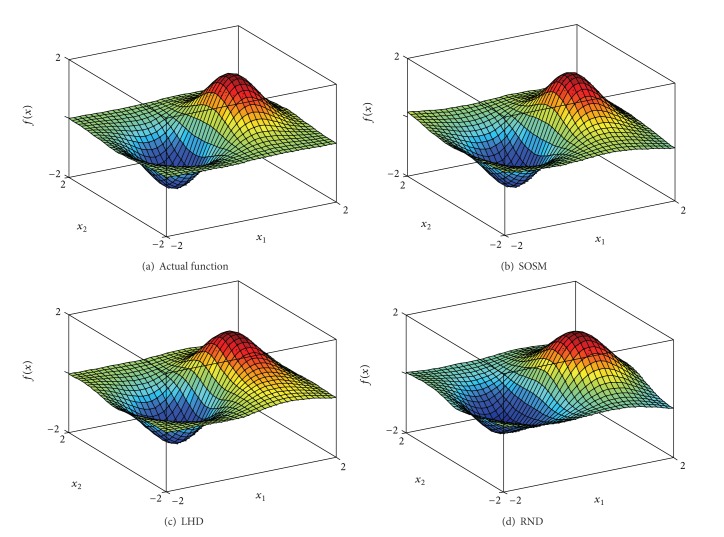
Function 2: actual and metamodel surface.

**Figure 13:**
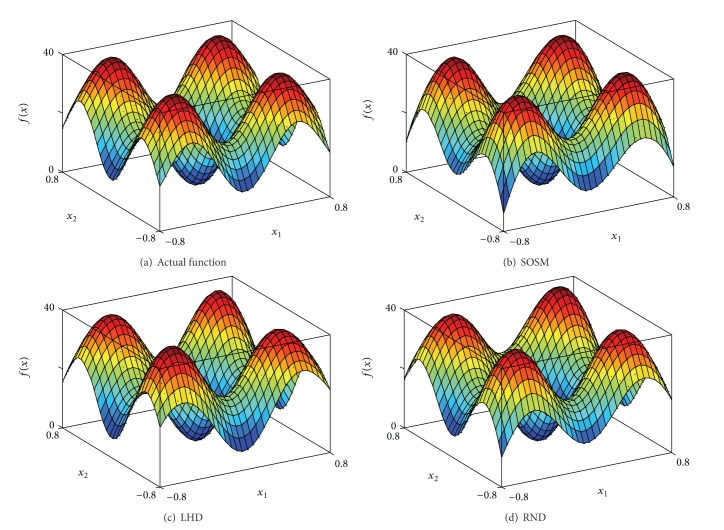
Function 3: actual and metamodel surface.

**Figure 14:**
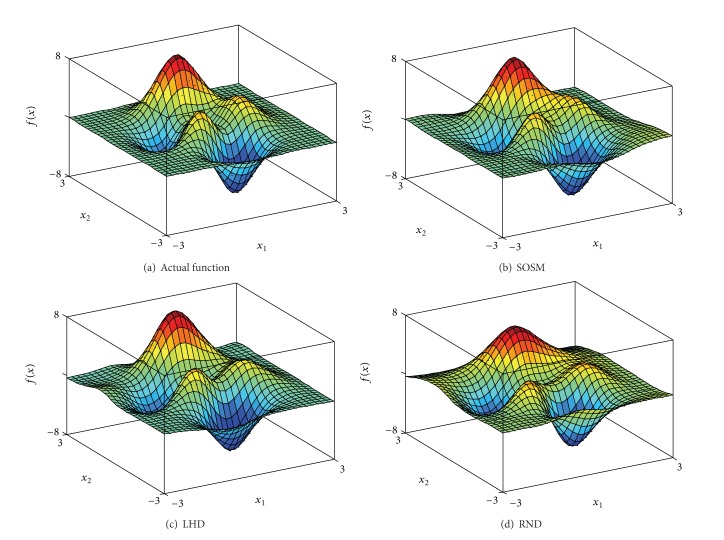
Function 4: actual and metamodel surface.

**Figure 15:**
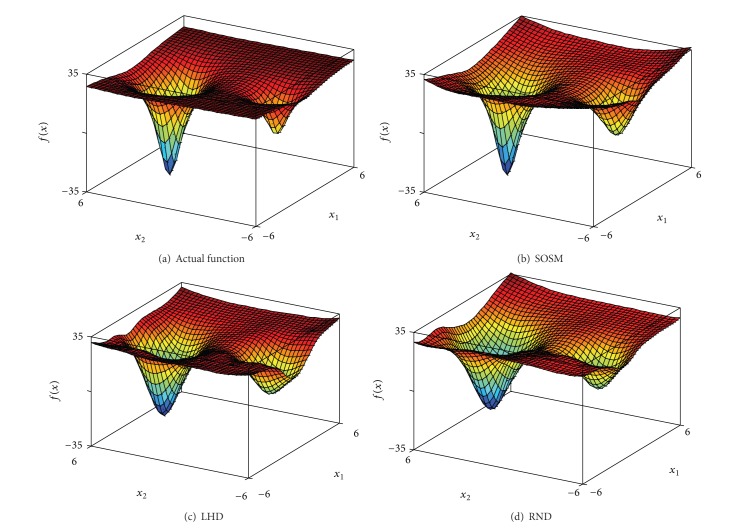
Function 5: actual and metamodel surface.

**Figure 16:**
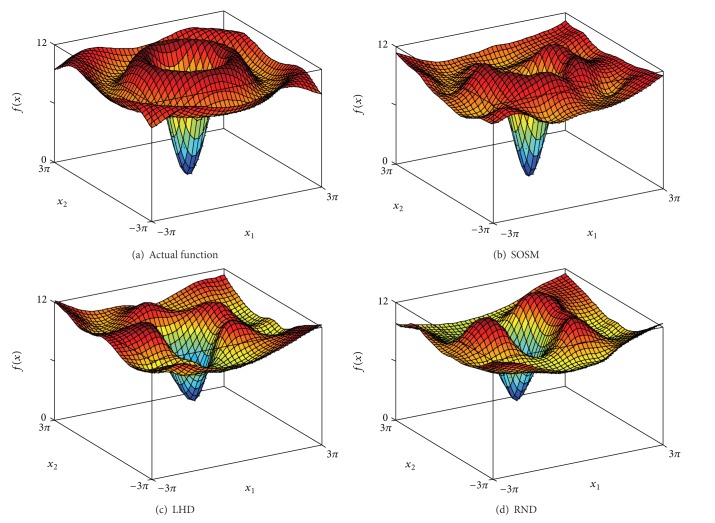
Function 6: actual and metamodel surface.

In order to indicate the effectiveness of SOSM in seeking global minimum points, the positions of global minimum points of metamodels for functions. 1–6 are shown in Figures 10(a)–10(f) separately. The red pentagon indicates the actual global minimum point. The black dots indicate the global minimum points of metamodels based on sampling method SOSM. The blue squares indicate the global minimum points of metamodels based on sampling method LHD. The magenta diamonds indicate the global minimum points of metamodels based on sampling method RND.  Figure 10 shows that the black dots are distributed densely in the center of red pentagon. Meanwhile, the blue squares and magenta diamonds are decentralized around the red pentagon and the difference between actual global minimum points and global minimum points of metamodels based on LHD or RND is mostly quite large. The above demonstrate that SOSM is superior to LHD and RND on global optimization. (a)–(d) in Figures 11, 12, 13, 14, 15, and 16 show the graphs of actual functions and of the associated metamodels based on three sampling methods separately. It can be observed intuitionally that the metamodels surface adopting SOSM method is smoother than LHD and RND and, furthermore, the global minima of metamodels based on SOSM are consistent with the actual global minima. The conclusions reached above are identified further by comparing the actual function surfaces to metamodels surfaces.

#### 4.4.2. Comparison of the Performance between SOSM and Previous Sequential Sampling Methods

In this part, three different sequential sampling methods including cross-validation sampling method (CV) [25], sequential sampling method proposed by Kitayama et al. [28] termed KSSM, and successive local enumeration sampling method (SLE) [29] are used to construct metamodels in comparison with those constructed by SOSM. Similarly, the accuracy of metamodels and global minimum of functions are obtained and managed. The accuracy measures RMSE and NRMSE and global minimum summarized in Table 5 are mean values. Note that a value of zero for both accuracy measures RMSE and NRMSE would indicate a perfect fit.

From Table 5, the RMSE and NRMSE of metamodels using sampling method SOSM are smaller than the sequential sampling methods CV and KSSM for functions 1–5. For function 6, the values are close. The SLE performs greatest. That is, metamodels based on sampling method SOSM may provide a better fit to actual functions than CV and KSSM. However, the sampling method SLE performs unsatisfactorily in terms of exploring global minimum. As seen in Table 5, on exploring global minimum ${\hat{f}}_{\min}$ of metamodels, SOSM is superior to the previous sequential sampling methods SLE through all numerical examples compared to the actual global minimum *f*_min⁡_. In addition, the sequential sampling methods CV and KSSM perform as great as SOSM on exploring the global minimum. In general, the sequential sampling method KSSM is the best, SOSM takes second place, and CV is the least. It can be concluded that the sequential sampling method SOSM proposed in this paper is the best choice considering the accuracy of metamodels and exploring global minimum.

**Figure 17:**
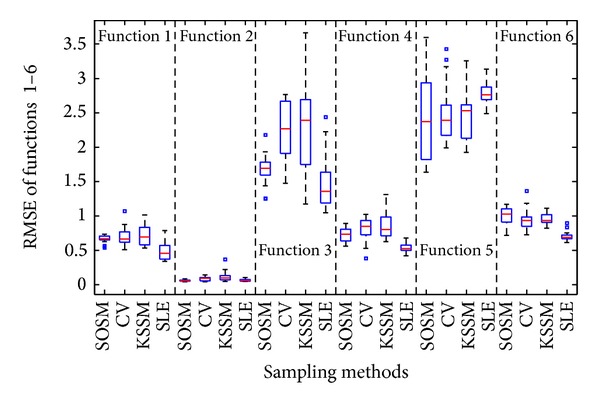
Assessment of metamodels: RMSE between SOSM and previous sequential sampling methods.

**Figure 18:**
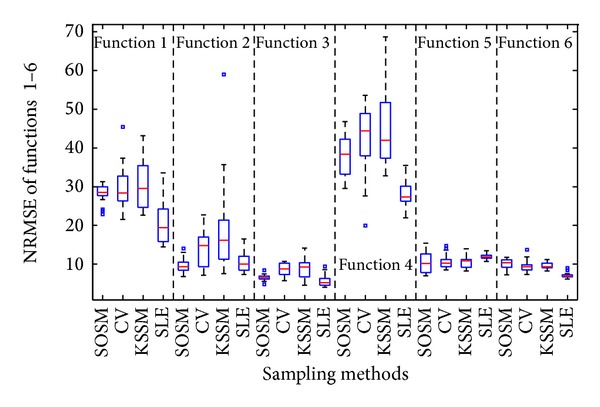
Assessment of metamodels: NRMSE between SOSM and previous sequential sampling methods.

**Figure 19:**
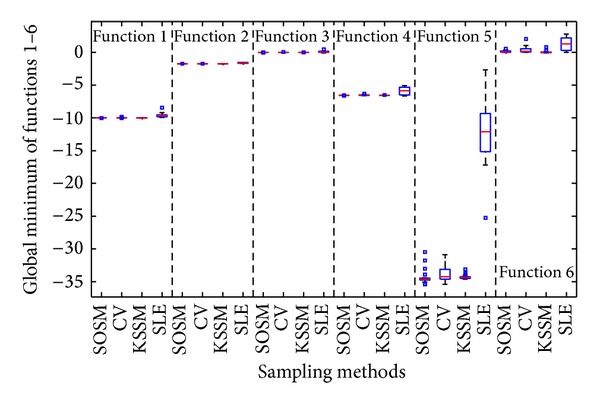
Assessment of metamodels: global minimum between SOSM and previous sequential sampling methods.

In order to represent the advantages and disadvantages of different sequential sampling methods adequately, the twenty times of metamodels accuracy results (RMSE and NRMSE) and global minimum *f*_min⁡_ with sequential sampling methods, that is, SOSM, CV, KSSM, and SLE, are illustrated in Figures 17–19 with the help of boxplot.

From the results shown in Figures 17 and 18, it is found that the median values of RMSE and NRMSE based on SOSM are smaller compared to CV and KSSM for functions 1–5. For function 6, the median values of RMSE and NRMSE are a little larger, but very close. Except for Function 5, the accuracy of metamodels constructed by SLE is the best. In addition, the box size of RMSE and NRMSE based on SOSM is shorter than CV and KSSM for functions 1–4. The box size of RMSE and NRMSE based on SLE is the shortest for all functions except for functions 1 and 3. A small size of the box suggests small standard deviation of results and low standard deviation of results suggests small uncertainties in the predictions and large standard suggests high uncertainty on the contrary. The points outside the horizontal lines indicate that the results of experiments are terrible. Above all, either mean values or median values and box sizes of results are taken into account, the accuracy of metamodels with sampling method SOSM is superior to sampling methods CV and KSSM. Certainly, the sampling method SLE performs greatest in terms of the accuracy of metamodels. However, SLE is not good at exploring the global minimum of functions.

Figure 19 depicts the boxplot of global minimum ${\hat{f}}_{\min}$ obtained for twenty numerical experiments. It is obvious from Figure 19 that the median value of SOSM, CV, and KSSM is probably equal to the actual global minimum *f*_min⁡_. Meanwhile, the small sizes of boxes imply small standard deviation, which is also reflected by small differences between the mean and median values. The standard deviation of global minimum is one of the important factors for evaluating the robustness of algorithm. Therefore, smaller standard deviation of results implies the robustness of the algorithm. It is clear from Figure 19 that the sequential sampling methods except SLE are robust sampling techniques under the parameter settings employed in this paper. The success rate is bad comparing the actual global minimum from Table 5 with the distribution of global minimum of metamodels based on sampling methods SLE from Figure 19. It is obvious that SOSM plays a perfect role in constructing the accurate metamodels and finding the global minimum of functions.

Six various sampling methods have been used to construct metamodels for six typical functions in this paper. It can be demonstrated that sequential sampling methods perform better and more efficient than one-stage sampling methods. Furthermore, sequential sampling technique allows engineers to control the sampling process. In general, one-stage sampling technique is not as good as sequential sampling methods for fitting the area where the global minimum locates. In a word, SOSM as a sequential sampling method proposed in this paper is the best choice considering the accuracy of metamodels and locating global minimum. In other words, SOSM is reliable in the whole fitting space and also good for fitting the area where the global minimum locates.

#### 4.4.3. Engineering Problem

**Figure 20:**
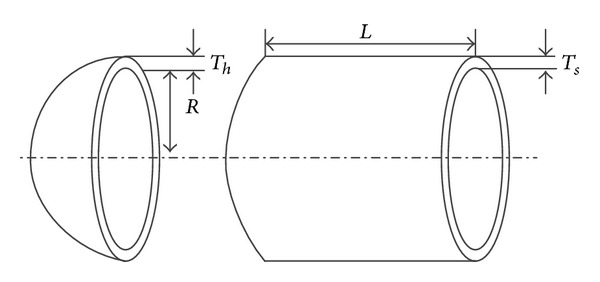
Diagram of pressure vessel design.

The validity of sequential sampling method SOSM proposed in this paper is tested by a typical mechanical design optimization problem involving four design variables, that is, pressure vessel design. This problem has been studied by many researchers [40–42]. The schematic of the pressure vessel is shown in Figure 20. In this case, a cylindrical pressure vessel with two hemispherical heads is designed for minimum fabrication cost. Four variables are identified: thickness of the pressure vessel *T*_*s*_, thickness of the head *T*_*h*_, inner radius of the pressure vessel *R*, and length of the vessel without heads *L*. In this case, the variable vectors are given (in inches) by

(15) $$\begin{array}{c} X=\left(T_{s},T_{h},R,L\right)=\left(x_{1},x_{2},x_{3},x_{4}\right). \end{array}$$

The objective function is the combined cost of materials, forming and welding of the pressure vessel. The mathematical model of the optimization problem is expressed as

(16) min⁡ f(X)=0.6224x1x3x4+1.7781x2x32+3.1661x12x4+19.84x12x3s.t.  g1(X)=−x1+0.0193x3≤0g2(X)=−x2+0.00954x3≤0g3(X)=−πx32x4−43πx33+129600≤0g4(X)=x4−240≤0.

The ranges of design variables *x*_1_ ∈ [0.0625,6.25], *x*_2_ ∈ [0.0625,6.25], *x*_3_ ∈ [62.5,125], and *x*_4_ ∈ [62.5,125] are used referring to the literature [40].

The problem formulated above is a simple nonlinear constrained problem. Now assuming that the objective and constraint functions defined by (16) are computation-intensive functions, hence, metamodels of functions are constructed by RBF using the sequential sampling method SOSM.

This problem has been solved by many researchers, including Cao and Wu [42], applying an evolutionary programming model. Kannan and Kramer [43] solved the problem using an augmented Lagrangian multiplier approach and Deb [44] using a genetic adaptive search.

The average values of optimal results from 50 runs are listed in Table 6 compared with the three results reported in the literature [42–44]. It can be seen from the table that the optimal solution in this paper is still about 2.7% superior to the best solution previously reported in the literature [44].

## 5. Conclusions

In this paper, the sequential optimization sampling method (SOSM) for metamodels has been proposed. The recently developed extended radial basis functions (E-RBF) are introduced as an approximated method to construct the metamodels. Combining the new sampling strategy SOSM with the extended radial basis functions, the design algorithm for building metamodels is presented. In the SOSM, the optimal sampling points of response surface are taken as the new sampling points in order to improve the local accuracy. In addition, new sampling points in the sparse region are required for better global approximation. To determine the sparse region, the density function constructed by the radial basis functions network has been applied. In the proposed algorithm, the response surface is constructed repeatedly until the terminal criterion, that is, the maximum number of sampling points *m*_max⁡_, is satisfied.

For the sake of examining the validity of the proposed SOSM sampling method, six typical mathematical functions have been tested. The assessment measures for accuracy of metamodels, that is, RMSE and NRMSE, are employed. Meanwhile, global minimum is introduced to judge the performance of sampling methods for metamodels. The proposed sampling method SOSM is successfully implemented and the results are analyzed comprehensively and thoroughly. In contrast to the one-stage sampling methods (LHD and RND) and sequential sampling methods (CV, KSSM, and SLE), the SOSM results in more accurate metamodels. Furthermore, SOSM is superior in exploring the global minimum of metamodels compared to the other five sampling methods.

The new sequential optimization sampling method SOSM has been proposed, which provides another effective way for generating sampling points to construct metamodels. It is superior in seeking global minimum compared to the previous sampling methods, and, meanwhile, can improve the accuracy of metamodels. Therefore, the sequential optimization sampling method SOSM significantly outperforms the previous sampling methods.

## Figures and Tables

**Table 1:** Commonly used basis functions.

Name	Radial function *r* = ||x−x_*i*_||_2_
Linear	*ϕ*(*r*) = *cr*
Cubic	*ϕ*(*r*) = (*r*+*c*)^3^
Thin-plate spline	*ϕ*(*r*) = *r* ^2^log⁡(*cr* ^2^)
Gaussian	*ϕ*(*r*) = exp⁡(−*cr* ^2^)
Multiquadric	*ϕ*(*r*) = (*r* ^2^+*c* ^2^)^1/2^

*c* is a constant.

**Table 2:** Nonradial basis functions *ψ*(*ξ*_*i*_^*j*^).

Region	Range of *ξ*_*i*_^*j*^	*ψ* ^*L*^	*ψ* ^*R*^	*ψ* ^*β*^
I	*ξ*_*i*_^*j*^ ≤ −*γ*	(−*ηγ* ^*η*−1^)*ξ*_*i*_^*j*^ + *γ* ^*η*^(1 − *η*)	0	*ξ*_*i*_^*j*^
II	−*γ* ≤ *ξ*_*i*_^*j*^ ≤ 0	(*ξ*_*i*_^*j*^)^*η*^	0	*ξ*_*i*_^*j*^
III	0 ≤ *ξ*_*i*_^*j*^ ≤ *γ*	0	(*ξ*_*i*_^*j*^)^*η*^	*ξ*_*i*_^*j*^
IV	*ξ*_*i*_^*j*^ ≥ *γ*	0	(*ηγ* ^*η*−1^)*ξ*_*i*_^*j*^ + *γ* ^*η*^(1 − *η*)	*ξ*_*i*_^*j*^

*γ*, *η* are prescribed parameters; refer to [32, 36].

**Table 3:** Numerical function and global minimum.

Number	Function	Design domain	Global minimum
1	*f*(*x*) = −10sin⁡⁡*c*(*x*_1_) · sin⁡⁡*c*(*x*_2_)	−2 ≤ *x* ≤ 2	*x* = (0,0), *f*_min⁡_ = −10

2	*f*(*x*) = 4*x*_1_ · *e* ^−*x*_1_^2^*l*^^−*x*_2_^2^^	−2 ≤ *x* ≤ 2	$x=(-\sqrt{2}/2,0),f_{\min}=-1.72$

3	*f*(*x*) = *x*_1_^2^*l*^^ + *x*_2_*l*__^2^ − 10∗[cos⁡(2*πx*_1_) + cos⁡(2*πx*_1_)] + 20	−0.8 ≤ *x* ≤ 0.8	*x* = (0,0), *f*_min⁡_ = 0

4	f(x)=3(1-x1)2·e-x12l-(x2+1)2-10(x15-x13-x25)f(x)=·e-x12-x22-13le-(x1+1)2-x22	−3 ≤ *x* ≤ 3	*x* = (0.228, −1.63), *f*_min⁡_ = −6.55

5	f(x)=-60/[1+(x1+1)2+(x2-3)2]-20/[1+(x1-1)2lf(x)=+(x2l-3)2]-30/[1+x12+(x2+4)2]+30	−6 ≤ *x* ≤ 6	*x* = (−0.97,3), *f*_min⁡_ = −34.63

6	f(x)=-10(sin⁡⁡x12+x22+eps/x12+x22+eps)+10,eps=10-15ll	−3*π* ≤ *x* ≤ 3*π*	*x* = (0,0), *f*_min⁡_ = 0

**Table 4:** Metamodel accuracy results for functions 1–6 between SOSM and one-stage sampling methods.

Number	SOSM			LHD			RND			*f*_min⁡_
	RMSE	NRMSE	${\hat{f}}_{\min}$	RMSE	NRMSE	${\hat{f}}_{\min}$	RMSE	NRMSE	${\hat{f}}_{\min}$	
1	0.663	28.214	−10.002	0.722	30.698	−9.137	0.817	34.766	−8.728	−10
2	0.061	9.798	−1.715	0.090	14.524	−1.611	0.122	19.659	−1.662	−1.715
3	1.674	6.464	−0.002	1.790	6.913	0.140	2.072	8.001	0.040	0
4	0.728	38.118	−6.556	0.810	42.456	−5.786	0.861	45.116	−5.374	−6.551
5	2.455	10.531	−34.243	3.353	14.380	−12.928	3.789	16.253	−9.907	−34.63
6	0.995	10.011	0.116	0.992	9.991	2.284	1.076	10.830	2.070	0

**Table 5:** Metamodel accuracy results for functions 1–6 between SOSM and previous sequential sampling methods.

Number	SOSM			CV [25]			KSSM [28]			SLE [29]			*f*_min⁡_
	RMSE	NRMSE	${\hat{f}}_{\min}$	RMSE	NRMSE	${\hat{f}}_{\min}$	RMSE	NRMSE	${\hat{f}}_{\min}$	RMSE	NRMSE	${\hat{f}}_{\min}$	
1	0.663	28.214	−10.002	0.699	29.707	−9.995	0.709	30.142	−10.017	0.493	20.951	−9.539	−10
2	0.061	9.798	−1.715	0.089	14.312	−1.715	0.120	19.343	−1.716	0.065	10.504	−1.622	−1.715
3	1.674	6.464	−0.002	2.261	8.732	0.000	2.305	8.902	−0.001	1.459	5.636	0.105	0
4	0.728	38.118	−6.556	0.813	42.601	−6.514	0.847	44.359	−6.552	0.533	27.899	−5.904	−6.551
5	2.455	10.531	−34.243	2.508	10.759	−33.794	2.466	10.575	−34.259	2.789	11.961	−12.212	−34.63
6	0.995	10.011	0.116	0.943	9.486	0.390	0.950	9.558	0.052	0.703	7.080	1.274	0

**Table 6:** Comparison of optimal results for the design of a pressure vessel.

Design variables	Cao and Wu [42]	Kannan and Kramer [43]	Deb [44]	This paper using SOSM
*x*_1_	1.000	1.125	0.9375	1.000
*x*_2_	0.625	0.625	0.5000	0.625
*x*_3_	51.1958	58.291	48.3290	41.523
*x*_4_	60.7821	43.690	112.6790	120.562
*f*(*X*)	7108.616	7198.042	6410.381	6237.402
